# Familiarity for Serious Mental Illness in Help-Seeking Adolescents at Clinical High Risk of Psychosis

**DOI:** 10.3389/fpsyt.2020.552282

**Published:** 2021-01-08

**Authors:** Michele Poletti, Silvia Azzali, Federica Paterlini, Sara Garlassi, Ilaria Scazza, Luigi Rocco Chiri, Simona Pupo, Andrea Raballo, Lorenzo Pelizza

**Affiliations:** ^1^Department of Mental Health and Pathological Addiction, Azienda Unità Sanitaria Locale-Istituto di Ricovero e Cura a carattere Scientifico (USL-IRCSS) di Reggio Emilia, Reggio Emilia, Italy; ^2^Department of Primary Care, Azienda Unità Sanitaria Locale (USL) di Parma, Parma, Italy; ^3^Anesthesia and Resuscitation Service, Azienda Ospedaliero-Universitaria di Parma, Parma, Italy; ^4^Division of Psychiatry, Department of Medicine, University of Perugia, Perugia, Italy; ^5^Center for Translational, Phenomenological and Developmental Psychopathology, Perugia University Hospital, Perugia, Italy; ^6^Department of Mental Health and Pathological Addiction, Azienda Unità Sanitaria Locale (USL) di Parma, Parma, Italy

**Keywords:** vulnerability, familiarity, early psychosis, early intervention, early detection, ultra-high risk (UHR) of psychosis, clinical high risk (CHR), adolescence

## Abstract

**Aim:** Ultrahigh-risk (UHR) individuals have an increased vulnerability to psychosis because of accumulating environmental and/or genetic risk factors. Although original research examined established risk factors for psychosis in the UHR state, these findings are scarce and often contradictory. The aims of this study were (a) to investigate the prevalence of severe mental illness (SMI) in family members of distinct subgroups of adolescents identified through the UHR criteria [i.e., non-UHR vs. UHR vs. first-episode psychosis (FEP)] and (b) to examine any relevant associations of family vulnerability and genetic risk and functioning deterioration (GRFD) syndrome with clinical and psychopathological characteristics in the UHR group.

**Methods:** Adolescents (*n* = 147) completed an *ad hoc* sociodemographic/clinical schedule and the Comprehensive Assessment of At-Risk Mental States to investigate the clinical status.

**Results:** More than 60% UHR patients had a family history of SMI, and approximately a third of them had at least a first-degree relative with psychosis or other SMI. A GRFD syndrome was detected in ~35% of UHR adolescents. GRFD adolescents showed baseline high levels of positive symptoms (especially non-bizarre ideas) and emotional disturbances (specifically, observed inappropriate affect).

**Conclusions:** Our results confirm the importance of genetic and/or within-family risk factors in UHR adolescents, suggesting the crucial need of their early detection, also within the network of general practitioners, general hospitals, and the other community agencies (e.g., social services and school).

## Introduction

In the last three decades, the early intervention in psychosis (EIP) paradigm has achieved increased consideration attention in the scientific community, generating focused programs of care within the mental health care network of different countries (1). Indeed, leaving patients with early psychosis untreated may have severe consequences in terms of quality of life, functioning, and health (e.g., treatment dropout, inpatient care, symptom severity), as well as in socioeconomic costs related to unemployment, long-term intervention, and poor outcomes of illness (2–4). However, to date, psychosis remains one of the most puzzling mental disorders, and our understanding of its etiopathological mechanisms is still far from being conclusive (5).

### Vulnerability to Psychosis and the “Ultrahigh-Risk” Paradigm

The most validated model to explain the etiology of psychosis is based on environmental and genetic risk factors and their interaction (in various modalities and at various levels), likely involving epigenetic mechanisms (6, 7). The evidence that many subjects who are at ultrahigh risk (UHR) of psychosis actually do not develop a full-blown psychotic episode seems to confirm this hypothesis, suggesting a complex interplay among genetic, neurodevelopmental, neuropsychological, sociocultural, and environmental factors in psychoses (6).

The detection of risk factors correlated with early psychosis is crucial to advance early identification of vulnerable subjects and to propose tailored interventions for young help-seeking individuals (8). Indeed, the delivery of specialized, evidence-based treatments as early as possible has become one of the current priorities for professionals involved in mental health care service network (9, 10).

Since its conceptualization, the UHR paradigm quickly became increasingly influential in the field of psychiatry (7). The UHR mental state is currently defined on the basis of three main inclusion criteria, which have been internationally validated: brief and limited intermittent psychotic symptoms (BLIPSs), attenuated psychotic symptoms (APSs), and genetic risk and functioning deterioration syndrome (GRFD) [for details, see (11)]. Specifically, APSs are subthreshold positive psychotic symptoms within the past 12 months. In the BLIPS group, criteria for psychosis are met for <7 days at a time and ceasing spontaneously (i.e., without antipsychotic medications). The GRFD syndrome is a state/trait risk condition in which the patient has a family history of psychosis (i.e., in first-degree relatives) or manifests a schizotypal personality disorder, along with low functioning sustained for at least 1 month. Accumulating findings have confirmed that young help-seekers meeting well-defined UHR psychometric criteria show an increased risk of developing psychosis (mostly schizophrenia spectrum disorders) within a relatively short period of time (12). Indeed, the psychosis conversion in people at UHR is most likely to occur within the first 24 months after the first contact to clinical services, with a risk of transition accumulating to 29% at 2 years (13). After this period, the speed of psychosis progression tends to plateau from the third year, reaching ~35% after 10 years (14). This risk is significantly greater than that reported in the general population: indeed, people at UHR have a 2-year relative risk of developing psychosis of 460, as compared to the general population (29%/0.063%) (7).

UHR subjects are likely to have an increased vulnerability to psychosis because of accumulating environmental and/or genetic risk factors (6). However, although several original research has explored the association of established risk factors for psychosis and the UHR state, the results are scarce and often contradictory, also with regard to the prevalence of severe mental illness (SMI) in family members of UHR individuals (15). As psychosocial dysfunction represents a common prodromal sign in UHR mental states, which exposes these young help-seeking individuals to social stigma and long-term interpersonal marginalization, reducing employment and economic opportunities (16), it is absolutely crucial to implement effective models of early detection of psychosis vulnerability as soon as possible within the mental health service network, especially because this “functional critical period” may be susceptible to change if effective interventions are provided (5).

Starting from this background, the first aim of the current study was to investigate the prevalence of SMI in family members of UHR adolescents compared to similar age group of help-seeking peers with first-episode psychosis (FEP) or not meeting both UHR and FEP criteria (11). Moreover, for better specifying the clinical profile of UHR adolescents with family prevalence of SMI (especially psychosis), we also examined any relevant associations of the presence of family members with SMI (and psychosis) with functioning and psychopathology in our UHR subgroup. Finally, for the same reasons, we also investigated any significant relationship of the presence of a GRFD syndrome (i.e., a specific clinical index of family history of psychosis in first-degree relatives of UHR subjects) with functioning, sociodemographic, clinical, and psychopathological characteristics in our UHR subsample. To the best of our knowledge, this is the first Italian study specifically developed to examine the prevalence of SMI in family members of UHR adolescent help-seekers recruiting within a specific EIP program, as well as the presence of a GRSD syndrome in adolescents with early psychosis.

## Materials and Methods

### Subjects

Participants were help-seeking adolescents who entered the “Reggio Emilia at Risk Mental States” (ReARMS) program [for details, see also (1)] between September 2012 and December 2018. All participants (*n* = 147) and their parents gave an informed consent prior to their inclusion in the research. Relevant local ethical approvals were sought for the study. This research has been also performed according to the Code of Ethics of the World Medical Association (Declaration of Helsinki) for experiments including humans. The data that support the findings of this study are available on request from the authors. The data are not publicly available because of privacy or ethical restrictions.

Inclusion criteria of the present research were (a) age 13–18 years, (b) specialist help-seeking, (c) UHR criteria defined by the CAARMS (the “Comprehensive Assessment of At-Risk Mental States”) (i.e., BLIPSs, APSs, and GRFD) [for details, see (11)], or (d) a duration of untreated psychosis (DUP: defined as the time interval between the beginning of psychotic features and the first antipsychotic treatment) (17) <2 years if a CAARMS-defined FEP is identified at the initial assessment (11). According to the EIP paradigm (4), a DUP <2 years is a crucial limit to begin an EIP intervention, being a shorter DUP correlated with better FEP outcomes (18, 19). In the ReARMS program, early detection of UHR/FEP help-seeking adolescents was a 2-step procedure (1, 20). The first was a screening step using the “Screening Schedule” for Psychosis (21), administered by general service staff members. The second step consisted of the CAARMS interview (to explore the presence of an UHR mental state, a first-episode psychosis or neither), within a baseline multidimensional assessment process also including an *ad hoc* clinical/sociodemographic schedule [for details, see also (1)]. UHR– individuals were excluded from the ReARMS protocol, but received specific information for an appropriate treatment (2).

Exclusion criteria were (a) previous Diagnostic and Statistical Manual of Mental Disorders, Fourth Edition, Text Revised (DSM-IV-TR) affective and non-affective psychoses (22); (b) past exposure to antipsychotics; (c) known intellectual disability (IQ <70); (d) neurological disease or any other medical illness with psychiatric features; and (e) current substance dependence, according to DSM-IV-TR criteria (22). Specifically, we considered past exposure to antipsychotics (i.e., before the ReARMS recruitment) as an equivalent of a previous psychotic episode, in accordance with CAARMS-defined FEP criteria, suggesting that the FEP threshold essentially corresponds to that at which antipsychotics would supposedly be started in the common clinical practice (11).

### Instruments and Measures

In the present research, the following instruments were used:

- An “*ad hoc* schedule” collecting specific clinical and sociodemographic information: i.e., gender, age, level of education, ethic group, mother tongue, employment status, prevalence of SMI in family members, *DSM-IV-TR* diagnosis, duration of untreated illness (DUI, defined as the time interval between the beginning of a marked psychopathological symptom and the first psychological/pharmacological intervention) (23), past hospitalization, previous specialist contact, previous suicide attempts [defined as a potentially injurious, self-inflicted behavior without a fatal outcome for which there was (implicit or explicit) evidence of intent to die] (24, 25), current substance abuse at the ReARMS enrollment, 1-year “dropout” rate, 1-year “psychosis transition” rate, 1-year CAARMS-defined psychometric criteria, and the specific ReARMS interventions provided to the users.
- CAARMS: a semistructured interview exploring several characteristics of the attenuated and full-blown psychotic psychopathology (i.e., positive symptoms, negative symptoms, disorganization, cognitive change, emotional disturbances, and general psychopathology), as well as the socio-occupational functioning [using the SOFAS (“Social and Occupational Functioning Assessment Scale”) module] [see (11), for details]. The CAARMS was administered by trained clinical psychologists and neuropsychiatrists [for details, see also (26)]. In the present research, we used the approved Italian version of the CAARMS (CAARMS-ITA) (27), which showed an excellent interrater reliability in Italian clinical samples of adolescents and young adults (26, 28).

In accordance with the DSM-IV-TR criteria (20), Axis I diagnoses were made using the Structured Clinical Interview for DSM-IV-TR Axis I Disorders (29). CAARMS UHR/FEP criteria (11) were used to separate the participants into the following three subsamples: (a) FEP sample; (b) UHR+ sample (i.e., BLIPSs, APSs, and GRFD), and (c) UHR– sample (i.e., those subjects who were below the CAARMS inclusion criteria). Finally, for better specifying the clinical profile of UHR adolescents with family history of SMI (especially psychosis), UHR+ participants were further dichotomized using the following criteria: (a) family history of SMI (i.e., considering all the degrees of family relationships), (b) presence of at least one first-degree relative with psychosis or other SMI, and (c) presence of a GRFD syndrome (i.e., alone or in comorbidity with APS or BLIPS condition). As suggested by Fulone et al. (30), for SMI, we expressly intended schizophrenia spectrum disorders or other related psychosis, bipolar disorder, and major depression.

### Procedures and Statistical Analysis

In the present research all participants underwent ReARMS program, a baseline multidimensional assessment process including the *ad hoc* clinical/sociodemographic schedule and the CAARMS interview (to explore the presence of an UHR mental state, a first-episode psychosis or neither) [for details, see also (1)]. UHR– individuals were excluded from the ReARMS protocol, but received specific advises for an appropriate treatment (2).

Depending on the severity of their symptoms and functioning decline, UHR and FEP adolescents were provided with a 5-year intervention package composed by pharmacological therapy and a multielement psychosocial treatment [including individual cognitive-behavioral therapy (CBT), psychoeducational sessions for family members, and a case management for an early rehabilitation], in accordance with the modern guidelines (31–33). Specifically, antipsychotics were avoided unless the UHR+ participants (a) were rapidly deteriorating in daily functioning, (b) were overwhelmed by psychotic symptoms, and (c) had an imminent risk of suicide or serious violence (2, 19). Atypical antipsychotics in low dose were typically used. Benzodiazepines and selective serotonin reuptake inhibitors were also administered to treat anxiety, insomnia, and depressive symptoms.

Collected data were analyzed using the 15.0 version of the Statistical Package for Social Science for Windows (34). Significance threshold was fixed at *p* = 0.05 for all two-tailed tests. Descriptive variables were represented using mean values ± standard deviation (if continuous parameters) or frequencies and percentages (if categorical parameters). As all explorations were not normally distributed (Kolmogorov–Smirnov-test with Lilliefors correction: *p* < 0.05) (34), non-parametric statistics were used. Specifically, intergroup comparisons on characteristics involving continuous variables were analyzed with the Kruskal–Wallis-test and the Mann–Whitney *U*-test (as appropriate). The χ^2^-test or the Fisher exact test (i.e., when 20% of expected frequency was ≤ 5 or any expected frequency was <1) was performed for categorical parameters. The Mann–Whitney *U*-test was also used as *post-hoc* procedure in comparisons of continuous variables within more than two subgroups. Finally, the Holm-Bonferroni correction method (35) was performed to counteract the problem of multiple comparisons. Specifically, sociodemographic, clinical, and psychopathological features were compared in UHR+ participants subsequently dichotomized using the following categories: (a) family history of SMI (i.e., considering all the degrees of family relationships), (b) presence of at least one first-degree relative with psychosis or other SMI, (c) presence of GRFD syndrome (i.e., alone or in comorbidity with APS or BLIPS condition).

## Results

A total of 147 adolescents [80 females (54.4%); 127 white adolescents (86.4%); mean age = 15.84 ± 1.67 years] consecutively entered the ReARM protocol from September 2012 to December 2018; of them, 96 (65.3%) youths were treated in the ReARMS program. As previously described [for details on characterization of young people with early psychosis who entered the ReARMS protocol, see also (2)], 11 adolescents were excluded because of exclusion criteria.

In the UHR+ subgroup [*n* = 51 (34.7% of the total sample)], 48 adolescents (94.1%) met the APS criteria, 2 (3.9%) met the BLIPS criteria, and only 1 met the GRFD criteria alone. Among the APS and BLIPS participants, 17 (34%) also met the GRFD criteria. At baseline, the most common diagnoses were represented by major depression (*n* = 23; 45.1%), anxiety disorders (*n* = 9; 17.6%), schizotypal personality disorder (*n* = 9; 17.6%), and obsessive–compulsive disorder (*n* = 4; 7.8%).

The FEP subgroup [*n* = 45 (30.6% of the total sample)] was composed of patients with DSM-IV-TR schizophrenia (*n* = 22; 48.9%), psychotic disorder not otherwise specified (*n* = 10; 22.2%), affective (major depressive or bipolar) psychosis (*n* = 9; 20.0%), and schizophreniform disorder (*n* = 4; 8.9%).

The remaining 51 adolescents (34.7% of the total sample) were under the CAARMS-defined UHR/FEP threshold and composed the UHR– subgroup. The most common diagnoses were represented by DSM-IV-TR depressive disorders (*n* = 22; 43.1%), non-schizotypal personality disorder (*n* = 18; 35.3%) (i.e., borderline, narcissistic, and avoidance personality disorder), and anxiety disorders (*n* = 11; 21.6%).

No intergroup differences in terms of gender, age, ethnic group, mother tongue, and years of education were observed (Table 1). Compared to UHR+, FEP adolescents showed a longer DUI. No intergroup differences were also found in terms of family history of SMI, as well as in first-degree relatives with psychosis or other SMI.

**Table 1:** Demographic and clinical characteristics of the total sample and the three subgroups.

**Variable**	**Total sample (*n* = 147)**	**UHR– (*n* = 51)**	**UHR+ (*n* = 51)**	**FEP (*n* = 45)**	**Statistics (χ^**2**^)**	***Post-hoc-*test (Mann–Whitney *U-*test)**
Gender (females)	80 (54.4%)	26 (51.0%)	31 (60.8%)	23 (51.1%)	1.27	—
Ethnic group (Caucasian)	127 (86.4%)	44 (86.3%)	46 (90.2%)	37 (82.2%)	1.29	—
Mother tongue (Italian)	139 (94.5%)	49 (96.1%)	50 (98.0%)	40 (88.9%)	1.57	—
Age at entry	15.84 ± 1.67	15.71 ± 1.72	15.57 ± 1.63	16.29 ± 1.60	5.51	—
Education (in years)	10.52 ± 1.68	10.67 ± 1.80	10.31 ± 1.57	10.60 ± 1.67	1.08	—
DUI (in weeks)	84.42 ± 57.08	81.00 ± 51.79	61.00 ± 40.57	113.77 ± 65.97	14.05*	FEP > UHR+^†^
Family history of SMI	89 (60.5%)	30 (58.8%)	31 (60.8%)	28 (62.2%)	0.12	—
First-degree relative with psychosis	21 (14.3%)	4 (7.8%)	8 (15.7%)	9 (20.0%)	3.01	—
First-degree relative with SMI	55 (37.4%)	21 (41.2%)	15 (29.4%)	19 (42.2%)	2.15	—

DUI, duration of untreated illness; FEP, patients with first-episode psychosis; UHR, ultrahigh-risk; UHR+, individuals who met CAARMS-defined UHR criteria; UHR–, individuals who did not meet CAARMS-defined UHR/FEP criteria; CAARMS, Comprehensive Assessment of At-Risk Mental States; SMI, severe mental illness. Frequencies and percentages, mean ± standard deviation, Kruskal–Wallis-test (χ^2^) and χ^2^-values are reported. ^*^p < 0.001; Holm-Bonferroni corrected p < 0.017.

### Family History of SMI and UHR+ Individuals: Clinical Profile

Among 31 UHR+ adolescents with family history of SMI [i.e., UHR+/F+ (60.8% of the UHR+ total group)], 30 met the APS criteria, and 1 met the BLIPS criteria at the baseline assessment; of them, 11 (35.5% of the UHR+/F+ total subgroup) also met the GRFD criteria.

Compared to UHR+ individuals without a family history of SMI (i.e., UHR+/F–), UHR+/F+ subjects showed a significantly lower CAARMS “Alogia” item subscore (Table 2). No other between-group difference in terms of baseline functioning, sociodemographic, clinical, and psychopathological characteristics was found. Similarly, no intergroup differences were observed in terms of frequency of specific ReARMS interventions provided to the UHR+ participants (i.e., antipsychotic medication, CBT, psychoeducational sessions for family members and case management oriented to an early recovery and rehabilitation).

**Table 2:** Sociodemographic, psychopathological, and clinical characteristics between UHR+ participants with and without family history of SMI.

**Variable**	**UHR+/F+** **(*n* = 31)**	**UHR+/F–** **(*n* = 20)**	**Statistics** **(χ^**2**^/*z*)**
Gender (females)	19 (61.3%)	12 (60.0%)	0.01
Ethnic group (Caucasian)	29 (93.5%)	17 (85.0%)	1.00
Mother tongue (Italian)	30 (96.8%)	20 (100.0%)	0.66
Employment status (student)	27 (87.1%)	18 (90.0%)	0.65
Age at entry	15.61 ± 1.67	15.50 ± 1.61	−0.85
Education (in years)	10.35 ± 1.58	10.25 ± 1.58	−1.34
DUI (in weeks)	60.15 ± 39.27	62.83 ± 45.02	−0.60
Past hospitalization	2 (6.5%)	3 (15.0%)	1.00
Previous specialist contact	16 (51.6%)	10 (50.0%)	0.13
Previous suicide attempt	6 (19.4%)	2 (10.0%)	0.80
Current substance abuse	1 (3.2%)	3 (15.0%)	2.33
1-year “dropout” rate	1 (3.2%)	3 (15.0%)	2.33
1-year “psychosis transition” rate	1 (4.5%)	3 (17.6%)	1.79
T1 APS criteria	11 (35.4%)	4 (20.0%)	2.84
T1 BLIPS criteria	2 (6.4%)	0 (0.0%)	1.63
T1 UHR– criteria	17 (54.8%)	13 (65.0%)	1.04
**CAARMS subscales**			
SOFAS	47.61 ± 9.56	48.50 ± 6.92	−0.56
Positive symptoms	10.13 ± 3.08	10.70 ± 3.67	−0.47
Cognitive change	4.19 ± 2.35	5.40 ± 2.37	−1.71
Emotional disturbance	5.16 ± 3.94	4.95 ± 2.95	−0.22
Negative symptoms	7.55 ± 4.11	8.15 ± 3.88	−0.65
Behavioral change	10.97 ± 5.10	10.10 ± 3.37	−0.14
Motor/physical changes	2.68 ± 2.87	4.15 ± 3.90	−1.02
General psychopathology	15.74 ± 5.82	16.10 ± 6.66	−0.45
**CAARMS items**			
Unusual thought content	2.58 ± 1.50	3.30 ± 1.45	−1.99
Non-bizarre ideas	2.52 ± 1.48	2.60 ± 1.23	−0.78
Perceptual abnormalities	2.90 ± 1.27	2.65 ± 1.72	−0.12
Disorganized speech	2.13 ± 1.52	2.15 ± 1.42	−0.20
Subjective cognitive change	2.68 ± 1.42	3.45 ± 1.39	−1.90
Observed cognitive change	1.52 ± 1.61	1.95 ± 1.39	−1.20
Subjective emotional disturbance	2.06 ± 1.91	2.45 ± 1.64	−0.84
Observed blunted affect	2.23 ± 1.78	2.00 ± 1.62	−0.39
Observed inappropriate affect	0.87 ± 1.14	0.50 ± 1.28	−1.67
Alogia	1.45 ± 1.67	2.60 ± 1.57	−2.11*
Avolition/apathy	2.87 ± 1.69	2.80 ± 1.70	−0.24
Anhedonia	3.23 ± 1.56	2.75 ± 1.58	−1.20
Social isolation	3.61 ± 1.54	3.75 ± 1.62	−0.54
Impaired role functioning	3.19 ± 1.66	3.00 ± 1.65	−0.22
Disorganizing/odd/ stigmatizing behavior	1.71 ± 1.72	1.80 ± 1.85	−0.13
Aggressive/dangerous behavior	2.45 ± 1.96	1.55 ± 1.43	−1.80
Subjective impaired motor functioning	0.35 ± 0.98	0.80 ± 1.36	1.45
Objective impaired motor functioning	0.68 ± 1.42	0.25 ± 0.91	1.18
Subjective impaired bodily sensation	0.42 ± 1.25	1.40 ± 1.72	−1.99
Subjective impaired autonomic functioning	1.23 ± 1.50	1.70 ± 1.89	−0.99
Mania	0.29 ± 0.90	0.35 ± 0.99	−0.52
Depression	3.61 ± 1.38	3.15 ± 1.63	−0.80
Suicidality/self-harm	1.77 ± 2.06	1.55 ± 1.47	−0.01
Mood swings/lability	1.39 ± 1.41	2.10 ± 1.86	−1.42
Anxiety	3.42 ± 1.49	3.35 ± 1.78	−0.45
Obsessive–compulsive symptoms	1.26 ± 1.71	1.50 ± 2.11	−0.21
Dissociative symptoms	1.16 ± 1.53	1.55 ± 1.64	−0.81
Subjective impaired tolerance to normal stress	2.84 ± 1.71	2.55 ± 1.73	−0.44
T0 exposure to antipsychotics	12 (38.7%)	6 (30.0%)	0.40
CBT	13 (41.9%)	12 (60.0%)	0.55
Family psychoeducation	12 (38.7%)	7 (35.0%)	0.69
Case management	13 (41.9%)	12 (60.0%)	0.55

DUI, duration of untreated illness; UHR, ultrahigh-risk; UHR+, individuals who met CAARMS-defined UHR criteria; UHR–, individuals who were below the CAARMS-defined UHR/FEP criteria; FEP, first-episode psychosis; CAARMS, Comprehensive Assessment of At-Risk Mental States; SMI, severe mental illness; UHR+/F+, UHR participants with a family history of SMI; UHR+/F–, UHR participants without a family history of SMI; APS, attenuated psychotic symptoms; BLIPS, brief and limited intermittent psychotic symptoms; SOFAS, Social and Occupational Functioning Assessment Scale; T0, baseline assessment; T1, 1-year assessment time; CBT, cognitive–behavioral therapy. Frequencies and percentages, mean ± standard deviation, Mann–Whitney U-test (z), and χ^2^-values are reported. ^*^Holm–Bonferroni corrected p < 0.025.

Among 15 UHR+ adolescents with at least a first-degree relative with psychosis or other SMI [i.e., UHR+/FDR+ (29.4% of the UHR+ total group)], 14 met the APS criteria and 1 met the BLIPS criteria at the baseline assessment; of them, 7 (46.7% of the UHR+/FDR+ total subgroup) also met the GRFD criteria.

Compared to UHR+ individuals without a first-degree relative with psychosis or other SMI (i.e., UHR+/FDR–), UHR+/FDR+ subjects showed a significantly lower CAARMS “alogia” and “suicidality/self-harm” item subscores (Table 3). No other intergroup differences in terms of baseline functioning and sociodemographic, clinical, and psychopathological features were found. Similarly, no between-group difference was reported in terms of frequency of specific ReARMS interventions provided to the UHR+ adolescents.

**Table 3:** Sociodemographic, psychopathological, and clinical characteristics between UHR+ participants with and without first-degree relatives with psychosis or other SMI.

**Variable**	**UHR+/FDR+** **(*n* = 15)**	**UHR+/FDR–** **(*n* = 36)**	**Statistics** **(χ**^**2**^**/*z*)**
Gender (females)	6 (40.0%)	25 (69.4%)	3.85
Ethnic group (Caucasian)	13 (86.7%)	33 (91.7%)	0.30
Mother tongue (Italian)	14 (93.3%)	36 (100.0%)	2.45
Employment status (student)	12 (80.0%)	33 (91.7%)	2.83
Age at entry	15.82 ± 1.61	15.26 ± 1.63	−1.23
Education (in years)	10.54 ± 1.55	10.04 ± 1.58	−1.28
DUI (in weeks)	56.87 ± 39.73	67.33 ± 42.42	−0.73
Past hospitalization	0 (0.0%)	5 (13.9%)	2.31
Previous specialist contact	10 (66.7%)	16 (44.4%)	2.09
Previous suicide attempt	0 (0.0%)	8 (22.2%)	3.95
Current substance abuse	1 (6.7%)	3 (8.3%)	0.04
1-year “dropout” rate	1 (6.7%)	3 (8.3%)	2.33
1-year “psychosis transition” rate	1 (6.7%)	3 (8.3%)	1.79
T1 APS criteria	7 (46.6%)	8 (22.2%)	2.84
T1 BLIPS criteria	1 (6.7%)	1 (2.7%)	1.63
T1 UHR– criteria	6 (40.0%)	24 (66.7%)	2.04
**CAARMS subscales**			
SOFAS	47.43 ± 10.05	48.61 ± 6.44	−0.48
Positive symptoms	10.29 ± 3.21	10.43 ± 3.49	−0.02
Cognitive change	4.39 ± 2.30	5.00 ± 2.56	−0.96
Emotional disturbance	5.25 ± 3.90	4.87 ± 3.15	−0.41
Negative symptoms	7.57 ± 4.14	8.04 ± 3.89	−0.58
Behavioral change	11.32 ± 5.24	9.78 ± 3.26	−0.83
Motor/physical changes	2.75 ± 3.00	3.87 ± 3.71	−0.85
General psychopathology	15.89 ± 6.00	15.67 ± 6.35	−0.22
**CAARMS items**			
Unusual thought content	2.33 ± 1.34	3.08 ± 1.46	−1.80
Non-bizarre ideas	3.20 ± 0.94	2.64 ± 1.61	−1.42
Perceptual abnormalities	2.80 ± 1.52	2.44 ± 1.32	−1.06
Disorganized speech	2.07 ± 1.58	2.17 ± 1.44	−0.08
Subjective cognitive change	2.47 ± 1.60	3.19 ± 1.35	−1.53
Observed cognitive change	1.67 ± 1.45	1.69 ± 1.58	−0.06
Subjective emotional disturbance	1.60 ± 1.80	2.47 ± 1.76	−1.54
Observed blunted affect	2.20 ±1.78	2.11 ± 1.70	−0.25
Observed inappropriate affect	0.80 ± 1.08	0.69 ± 1.26	−0.62
Alogia	1.07 ± 1.39	2.25 ± 1.73	−2.21*
Avolition/apathy	2.93 ± 1.49	2.81 ± 1.69	−0.52
Anhedonia	3.33 ± 1.40	2.92 ± 1.64	−0.63
Social isolation	3.60 ± 1.45	3.69 ± 1.62	−0.53
Impaired role functioning	3.20 ± 1.37	3.08 ± 1.76	−0.21
Disorganizing/odd/ stigmatizing behavior	1.73 ± 1.98	1.75 ± 1.68	−0.25
Aggressive/dangerous behavior	2.40 ± 2.06	1.97 ± 1.71	−0.84
Subjective impaired motor functioning	0.47 ± 1.25	0.56 ± 1.13	−0.57
Objective impaired motor functioning	0.67 ± 1.59	0.44 ± 1.11	−0.36
Subjective impaired bodily sensation	0.13 ± 0.52	1.08 ± 1.71	−2.05
Subjective impaired autonomic functioning	0.87 ± 1.30	1.64 ± 1.76	−1.55
Mania	0.60 ± 1.24	0.19 ± 0.75	−1.22
Depression	3.33 ± 1.45	3.47 ± 1.52	−0.54
Suicidality/self-harm	0.87 ± 1.51	2.03 ± 1.87	−2.27*
Mood swings/lability	1.67 ± 1.34	1.67 ± 1.74	−0.18
Anxiety	2.93 ± 1.75	3.58 ± 1.50	−1.45
Obsessive–compulsive symptoms	1.40 ± 1.64	1.33 ± 1.97	−0.48
Dissociative symptoms	1.00 ± 1.60	1.44 ± 1.56	−0.95
Subjective impaired tolerance to normal stress	2.40 ± 1.80	2.86 ± 1.67	−1.70
T0 exposure to antipsychotics	6 (40.0%)	12 (33.3%)	0.21
CBT	8 (53.3%)	17 (47.2%)	0.46
Family psychoeducational	7 (46.6%)	12 (33.3%)	0.01
Case management	7 (46.6%)	18 (50.0%)	1.89

DUI, duration of untreated illness; UHR, ultrahigh risk; UHR+, individuals who met CAARMS-defined UHR criteria; UHR–, individuals who were below the CAARMS-defined UHR/FEP criteria; FEP, first-episode psychosis; CAARMS, Comprehensive Assessment of At-Risk Mental States; SMI, severe mental illness; UHR+/FDR+, UHR participants with at least a first-degree relative with psychosis or other SMI; UHR+/FDR–, UHR participants without first-degree relatives with psychosis or other SMI; APS, attenuated psychotic symptoms; BLIPS, Brief and Limited Psychotic Symptoms; SOFAS, Social and Occupational Functioning Assessment Scale; T0, baseline assessment; T1, 1-year assessment time; CBT, cognitive–behavioral therapy. Frequencies and percentages, mean ± standard deviation, Mann–Whitney U-test (z), and χ^2^-values are reported. *Holm–Bonferroni corrected p < 0.025.

### GRFD Syndrome in UHR+ Individuals: Clinical Profile

Among 51 UHR+ participants, 18 (35.2% of the UHR+ total group) met the CAARMS-defined GRFD criteria (i.e., UHR+/GRFD+); of them, 17 individuals (94.4% of the total GRFD subgroup) showed a co-occurrence of GRFD syndrome with APS or BLIPS criteria.

Compared to UHR+ subjects not meeting the GRFD criteria (i.e., UHR+/GRFD–), UHR+/GRFD+ individuals showed a significantly higher CAARMS “positive symptoms” and “emotional disturbance” subscale scores, as well as higher “non-bizarre ideas” and “observed inappropriate affect” item subscores (Table 4). No other intergroup differences in terms of baseline functioning and sociodemographic, clinical, and psychopathological characteristics were observed. Similarly, no between-group differences were found in terms of frequency of specific ReARMS interventions provided to the UHR+ participants.

**Table 4:** Sociodemographic, psychopathological, and clinical characteristics between UHR+ participants meeting or not meeting CAARMS-defined GRFD criteria.

**Variable**	**UHR+/GRFD+** **(*n* = 18)**	**UHR+/GRFD– (*n* = 33)**	**Statistics** **(χ^**2**^/*z*)**
Gender (females)	11 (61.1%)	20 (60.6%)	0.01
Ethnic group (Caucasian)	15 (83.3%)	31 (93.3%)	1.48
Mother tongue (Italian)	17 (94.4%)	33 (100.0%)	1.87
Employment status (student)	15 (83.3%)	30 (90.9%)	1.96
Age at entry	16.06 ± 1.35	15.30 ± 1.72	−1.58
Education (in years)	10.67 ± 1.37	10.12 ± 1.65	−1.12
DUI (in weeks)	49.62 ± 32.71	66.92 ± 43.54	−1.20
Past hospitalization	2 (11.1%)	3 (9.1%)	0.05
Previous specialist contact	11 (61.1%)	15 (45.5%)	1.14
Previous suicide attempt	1 (5.6%)	7 (21.29%)	2.16
Current substance abuse	1 (5.6%)	3 (9.1%)	0.20
1-year “dropout” rate	1 (5.6%)	3 (9.1%)	0.20
1-year “psychosis transition” rate	2 (13.3%)	2 (8.3%)	0.25
T0 APS criteria	16 (88.9%)	32 (97.0%)	1.37
T0 BLIPS criteria	1 (5.6%)	1 (3.0%)	0.20
T1 APS criteria	4 (22.2%)	11 (35.4%)	1.43
T1 BLIPS criteria	1 (5.6%)	1 (3.0%)	0.12
T1 UHR– criteria	7 (38.9%)	20 (60.6%)	2.06
**CAARMS subscales**			
SOFAS	48.56 ± 5.19	47.64 ± 9.99	−0.29
Positive symptoms	11.94 ± 2.75	9.48 ± 3.29	−2.51*
Cognitive change	5.17 ± 2.18	4.39 ± 2.52	−1.26
Emotional disturbance	6.50 ± 3.03	4.30 ± 3.62	−2.21*
Negative symptoms	7.94 ± 3.37	7.70 ± 4.35	−0.09
Behavioral change	11.17 ± 5.06	10.33 ± 4.20	−0.31
Motor/physical changes	2.89 ± 3.56	3.45 ± 3.27	−0.89
General psychopathology	15.11 ± 6.50	16.30 ± 5.93	−0.62
**CAARMS items**			
Unusual thought content	3.22 ± 1.35	2.67 ± 1.57	−1.16
Non-bizarre ideas	3.44 ± 0.98	2.45 ± 1.56	−2.25*
Perceptual abnormalities	2.78 ± 1.55	2.42 ± 1.27	−0.95
Disorganized speech	2.50 ± 1.25	2.50 ± 1.25	−1.40
Subjective cognitive change	3.22 ± 1.31	2.85 ± 1.52	−0.65
Observed cognitive change	1.94 ± 1.39	1.55 ± 1.60	−0.98
Subjective emotional disturbance	2.50 ± 1.42	2.06 ± 1.98	−0.79
Observed blunted affect	2.72 ± 1.45	1.82 ± 1.78	−1.84
Observed inappropriate affect	1.28 ± 1.45	0.42 ± 0.90	−2.28*
Alogia	1.94 ± 1.63	1.88 ± 1.78	0.34
Avolition/apathy	3.06 ± 1.51	2.73 ± 1.68	−0.69
Anhedonia	2.94 ± 1.05	3.09 ± 1.81	−1.25
Social isolation	3.94 ± 1.26	3.52 ± 1.70	−0.55
Impaired role functioning	2.94 ± 1.80	3.21 ± 1.58	−0.37
Disorganizing/odd/stigmatizing behavior	2.00 ± 1.64	1.61 ± 1.82	−1.03
Aggressive/dangerous behavior	2.28 ± 2.14	2.00 ± 1.64	−0.59
Subjective impaired motor functioning	0.50 ± 0.98	0.55 ± 1.25	−0.21
Objective impaired motor functioning	0.33 ± 0.84	0.61 ± 1.43	−0.28
Subjective impaired bodily sensation	0.56 ± 1.38	0.94 ± 1.60	−1.00
Subjective impaired autonomic functioning	1.50 ± 2.00	1.36 ± 1.47	−0.01
Mania	0.22 ± 0.73	0.36 ± 1.02	−0.18
Depression	3.67 ± 1.49	3.30 ± 1.49	−0.88
Suicidality/self-harm	1.28 ± 1.53	1.91 ± 1.97	−1.03
Mood swings/lability	1.89 ± 1.71	1.55 ± 1.58	−0.76
Anxiety	3.28 ± 1.71	3.45 ± 1.54	−0.07
Obsessive–compulsive symptoms	1.11 ± 1.57	1.48 ± 2.02	−0.39
Dissociative symptoms	1.39 ± 1.65	1.27 ± 1.55	−0.18
Subjective impaired tolerance to normal stress	2.28 ± 1.71	2.97 ± 1.69	−1.42
T0 exposure to antipsychotics	7 (38.9%)	11 (33.3%)	0.16
CBT	10 (55.5%)	15 (45.5%)	0.07
Family psychoeducation	5 (27.7%)	14 (42.2%)	2.31
Case management	8 (44.4%)	17 (51.5%)	1.23

DUI, duration of untreated illness; UHR, ultrahigh risk; UHR+, individuals who met CAARMS-defined UHR criteria; UHR–, individuals who were below the CAARMS-defined UHR/FEP criteria; FEP, first-episode psychosis; CAARMS, Comprehensive Assessment of At-Risk Mental States; GFRD, genetic risk and functioning deterioration syndrome; UHR+/GRFD+, UHR participants who met CAARMS-defined GRFD criteria; UHR+/GRFD–, UHR participants who did not meet CAARMS-defined GRFD criteria; APS, attenuated psychotic symptoms; BLIPS, Brief and Limited Intermittent Psychotic Symptoms; SOFAS, Social and Occupational Functioning Assessment Scale; T0, baseline assessment; T1, 1-year assessment time; CBT, cognitive–behavioral therapy. Frequencies and percentages, mean ± standard deviation, Mann–Whitney U-test (z), and χ^2^-values are reported. ^*^Holm–Bonferroni corrected p < 0.025.

## Discussion

The first aim of the present research was to examine the prevalence of SMI in family members of distinct help-seeking subsamples of adolescents identified through the UHR criteria (i.e., UHR+ vs. FEP vs. UHR–). Although no statistically significant intergroup-differences were found in terms of family load for SMI, more than 60% of our UHR+ and FEP adolescents showed a general family history of SMI, and approximately a third of them (with percentages ranging from 29.4% in UHR+ subjects to 42.2% in FEP patients) had at least a first-degree relative with psychosis or other SMI. As expected, these findings confirm the epidemiological burden of a family history of SMI in the prodromal phase of psychosis and at the onset of illness, already in adolescence, consistently with the very few results reported in the current literature (15, 36–38).

However, our UHR+ adolescents with a family history of SMI (specifically those with a first-degree relative with psychosis or other SMI) showed lower levels of alogia, self-harm, and suicidality (i.e., suicidal thoughts and behaviors). These results seem to suggest that the experience of a family psychiatric suffering and the custom of living with people with an SMI could increase the personal ability to find words to express a request for specialist help and to describe their symptoms, as well as to support their hope and future projects. Moreover, this also confirms the absolute need for clinical attention for children and adolescents with family members with SMI (39) and the crucial importance of psychotherapeutic and psychoeducational interventions to support their coping skills, their resilience, and their quality of life (40).

In the present research, a GRFD syndrome was detected in ~35% of UHR+ adolescents, mostly (i.e., almost in 95% of cases) in co-occurrence with APS or BLIPS criteria. This result is slightly higher than that reported in the current literature (6, 41) and confirms the very low incidence of a GRFD syndrome alone in adolescent help-seeking populations attending specialist mental health services offering dedicated EIP programs (2, 7). Hence, there is a specific need to diffusely spread the early identification of psychosis in all the community services (e.g., school and social agencies, general practitioners, general hospital, emergency room), going beyond the boundaries of mental health centers and emphasizing the crucial attention to be paid to children and adolescent with a family history of SMI (40, 41) or with schizotypal personality traits (42, 43), together with a socio-occupational functioning decline. From a psychopathological point of view, in the present study, GRFD adolescents showed baseline high levels of positive symptoms (especially, non-bizarre ideas) and emotional disturbances (specifically, observed inappropriate affect). These specific clinical features may be useful for an early characterization of adolescents with a genetic risk of psychosis and an incipient functioning deterioration, also in developing specific screening test.

### Limitations

A first methodological limitation of the current research is the relatively small sample size. Therefore, further studies on larger populations of both UHR and FEP adolescents are needed.

Second, our sample was recruited within a specific EIP program. Thus, our results cannot be generalized outside UHR/FEP-enriched populations.

Third, future studies to better specify and deepen the clinical profile of UHR adolescents (e.g., non-bizarre ideas or emotional disturbances) meeting GRFD criteria are also recommended.

### Conclusions

This is the first Italian study specifically developed to investigate the prevalence of SMI and in family members of adolescent help-seekers recruited within a specific EIP program, as well as the presence of a GRFD syndrome in Italian youths with early psychosis. Our results confirm the importance of family load and genetic risk factors in young people at UHR of psychosis (as well as in FEP adolescents), suggesting the need of their early detection, already within the network of general practitioners, general hospitals, and the other community agencies (e.g., social services and school). Moreover, this clinical attention becomes even more crucial because adolescents receiving treatment in CAMHS are at elevated risk of falling through the child-adult service gap as they cross the transition boundary between services (i.e., from child–adolescent mental health services to adult mental health services) (1). Bridging this gap might be achievable through a framework shift that incorporates the full continuum of service response within a prevention and promotion framework for youth mental health (44).

## Data Availability Statement

The raw data supporting the conclusions of this article will be made available by the authors, without undue reservation.

## Ethics Statement

The studies involving human participants were reviewed and approved by AVEN (Area Vasta Emilia Nord) Ethics Committee (protocol 36102/2019). Written informed consent to participate in this study was provided by the participants' legal guardian/next of kin.

## Author Contributions

LP and AR designed the study and conducted the main data analysis. SA, FP, SG, IS, and LC collected data. LP and SP managed the literature. MP, LP, and AR drafted the manuscript. All authors read and approved the final version of the manuscript.

## Conflict of Interest

The authors declare that the research was conducted in the absence of any commercial or financial relationships that could be construed as a potential conflict of interest.
